# Efficacy of Two Moroccan *Cistus* Species Extracts against Acne Vulgaris: Phytochemical Profile, Antioxidant, Anti-Inflammatory and Antimicrobial Activities

**DOI:** 10.3390/molecules28062797

**Published:** 2023-03-20

**Authors:** Maryem Bouabidi, Federica Lina Salamone, Chemseddoha Gadhi, Hafida Bouamama, Antonio Speciale, Giovanna Ginestra, Luana Pulvirenti, Laura Siracusa, Antonia Nostro, Mariateresa Cristani

**Affiliations:** 1 Laboratory of Sustainable Development and Health Research (LRDDS), Faculty of Sciences and Technology, Cadi Ayyad University, 549 Bd Abdelkrim Al Khattabi, Marrakesh 40000, Morocco; 2 Dipartimento di Scienze Chimiche, Biologiche, Farmaceutiche ed Ambientali, Università di Messina, Viale F. Stagno D’Alcontres 31, 98166 Messina, Italy; 3 Laboratory of Agri-Food, Biotechnology and Valorization of Plant Bioresources, Faculty of Sciences Semlalia, Cadi Ayyad University, Bd. Prince My Abdellah, B.P. 2390, Marrakesh 40000, Morocco; 4 Istituto di Chimica Biomolecolare del Consiglio Nazionale delle Ricerche (ICB-CNR), Via Paolo Gaifami, 18, 95126 Catania, Italy

**Keywords:** traditional remedies, *Cistus* spp., acne vulgaris, HPLC-ESI-/MS, *Propionibacterium acnes*, polyphenols, cytotoxicity

## Abstract

Background: The genus *Cistus* L. (Cistaceae) includes several medicinal plants growing wild in the Moroccan area. Acne vulgaris (AV) is a chronic skin disorder treated with topical and systemic therapies that often lead to several side effects in addition to the development of antimicrobial resistance. Our study aimed to investigate the bioactivity of extracts of two Moroccan *Cistus* species, *Cistus laurifolius* L. and *Cistus salviifolius* L., in view of their use as potential coadjuvants in the treatment of mild acne vulgaris. Methods: Targeted phytochemical profiles obtained by HPLC-DAD and HPLC-ESI/MS analyses and biological activities ascertained by several antioxidants in vitro chemical and cell-based assays of the leaf extracts. Moreover, antimicrobial activity against Gram-positive and Gram-negative bacteria, and *Candida albicans* was evaluated. Results: Analyses revealed the presence of several polyphenols in the studied extracts, mainly flavonoids and tannins. *Cistus laurifolius* L. and *Cistus salviifolius* L. possessed good biological properties and all extracts showed antibacterial activity, particularly against *Staphylococcus aureus*, *S. epidermidis,* and *Propionibacterium acnes*, identified as the main acne-causing bacteria. Conclusion: The results suggest that examined extracts are promising agents worthy of further studies to develop coadjuvants/natural remedies for mild acne treatment.

## 1. Introduction

Skin is the largest organ and the main interface of the human body with the external environment. The skin condition largely depends on the overall state of health of individuals and acne vulgaris (AV) is one of the most common and severe skin disorders. Acne lesions are typically classified as non-inflammatory (open and closed comedones) or inflammatory, including papules and pustules or nodules [1,2]. It is mainly seen on the subjects’ face, the upper part of their chest, and on their back [3]; however, these disorders have a negative impact on the mental health, self-esteem, and quality of life of individuals [4,5].

The clinical and histological features define acne as a chronic inflammatory disease of the pilosebaceous unit [6]. Therefore, AV is pathophysiologically triggered by an increased sebum’s production, and follicular hyperkeratinization leading to clogging of the sebaceous/oil duct and colonization of bacteria, especially the *Propionibacterium acnes* (recently called *Cutibacterium acnes*), a bacterium implicated in complex inflammatory processes involving both innate and acquired immunity [2,5,7].

Most cases of acne do not require specific medical evaluation, although a medical workup could sometimes be worthwhile. Conventional topical therapies for mild AV mainly include retinoids, benzoyl peroxide, azelaic acid, and/or drug combinations [8]. In severe AV cases, isotretinoin, oral antibiotics, topical antimicrobials, and hormonal therapy agents are the first line of treatment. Nevertheless, conventional treatments may result in bacterial resistance, intolerable adverse effects, high treatment costs, and long-term therapy [3,9,10]. For instance, teratogenicity is one of the most serious potential side effects of isotretinoin [11].

Nowadays, researchers are increasingly interested in using medicinal plants and plant-derived products as co-adjuvant therapy for mild AV treatment. This interest is due to the recognition of their good pharmacological properties, lower adverse effects, and relatively low cost. Known as the rockroses, *Cistus* L. (Cistaceae L.) is among the genus that comprises many interesting medicinal species mainly widespread in the Mediterranean region, and twelve species belong to the Moroccan flora [12]. The evergreen plants of *Cistus* have been used as remedies in different folk and traditional medicines since ancient times; various *Cistus* species are still used in Italy, Morocco, Greece, Spain, and Turkey as general remedies and have contributed to the treatment of several health issues [13]. Phytochemical studies on different *Cistus* species have revealed the presence of several phenolic compounds, mainly phenolic acids, flavonoids, and tannins derivatives [14,15], that are probably involved in the biological activities of the plant, essentially in oxidative stress and inflammation prevention [16,17].

The overriding idea of our study was to find proof of the bioactivity of extracts of two Moroccan *Cistus* species, assuming they might be potential plant-derived products used as natural remedies for mild acne treatment. Thus, our study aimed to compare the phytochemical profiles, as well as to investigate the biological activities and the anti-acne potential of leaves hydroalcoholic (crude) extracts obtained from the two *Cistus* species: *Cistus laurifolius* L. (CLLE) and *Cistus salviifolius* L. (CSLE) and their polyphenolic fractions (CLLE PF and CSLE PF).

## 2. Results

### 2.1. Phytochemicals 

#### 2.1.1. Colorimetric Analysis

The chemical composition revealed that *Cistus laurifolius* L. and *Cistus salviifolius* L. extracts are rich in total phenolic compounds. The results reported in Table 1 show that both extracts, and especially their polyphenol fractions, possess a high quantity of flavonoids, mainly flavonols.

#### 2.1.2. HPLC-DAD and HPLC-ESI-/MS Based Secondary Metabolic Profiles

The detailed composition of CLLE and CSLE together with the corresponding phenolic fractions CLLE PF and CSLE PF are reported in Table 2. The crude extracts are characterized by the presence of many polyphenols belonging to three main biochemical classes: flavonoids, hydroxycinnamic acids, and tannins. Among the 36 peaks detected and tentatively identified, numerous methylated flavonoids stand out, such as methyl and di-methyl derivatives of flavonols quercetin and kaempferol as well as those of flavones apigenin and luteolin (Table 2 and Table S1 and Figure S1).

### 2.2. Antioxidant Activity

In most electron transfer (ET)-based assays, the antioxidant reaction is simulated with a suitable redox-potential agent. The antioxidants react with a fluorescent or colored oxidizing agent instead of peroxyl radicals. ET-based assays include ferric reducing/antioxidant power (FRAP) assay, ABTS assay, DPPH assay and others assays [18]. Considering the hypothesis that suggests the oxygen free radicals generated by neutrophils in the follicular wall could be involved in the pathogenesis of acne [19], the evaluation of the antioxidant potential of our extracts seemed necessary. 

The measurement of free radical scavenging indicated that all extracts possessed a noteworthy activity (Table 3). The higher DPPH scavenging activity was demonstrated for the polyphenolic fraction of CSLE (mmol TE/mg 2.593 ± 0.572) followed by the polyphenolic fraction of CLLE (mmol TE/mg 1.284 ± 0.327) (Table 3). The ABTS assay reveals that all extracts were equally efficient in reducing the ABTS cations. Furthermore, all extracts possess good ferric trichloride reduction capacity, particularly the CLSE PF extract. 

### 2.3. Anti-Inflammatory Activity 

**Figure 1:**
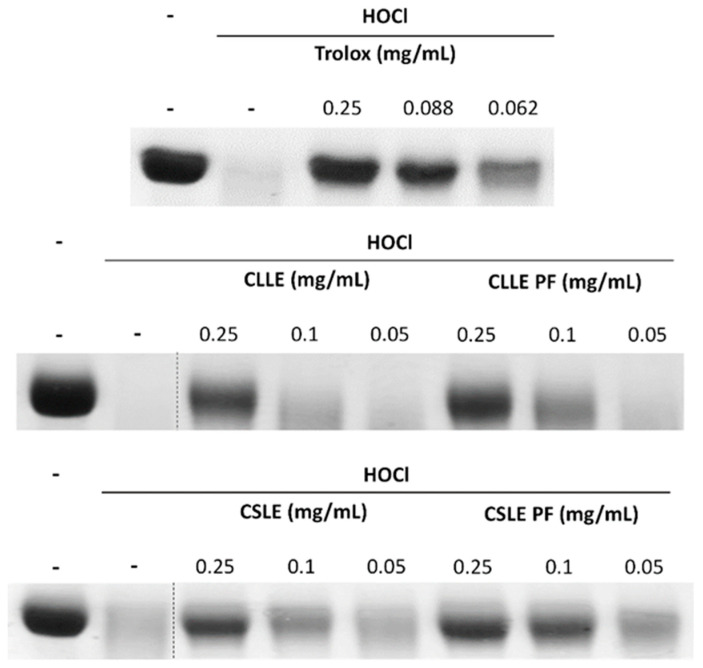
Inhibition of HClO-induced BSA degradation by *C. laurifolius* L. and *C. salviifolius* L. crude extracts and polyphenolic fractions. Image representative of three independent experiments. Dashed vertical black lines indicate removal of irrelevant lanes.

The HClO-induced BSA denaturation was carried out to investigate the anti-inflammatory capability of the extracts under study. It is well-known that HClO reacts with a wide variety of biological molecules producing tissue damage that impacts the function of biological systems, and that protein degradation is a well-documented cause of inflammatory diseases [20]. Among the tested samples, the extracts of *C. salviifolius* L. possessed the higher capacity to protect albumin against HClO-induced degradation comparable to Trolox used as standard (Figure 1 and Table 4).

### 2.4. Antimicrobial Activity

The MIC values analysis (Table 5) showed that the plant extracts possessed an inhibitory effect against tested strains. However, they demonstrated more efficacy against Gram-positive bacteria than against Gram-negative bacteria and *C. albicans* ATCC 10231. All *Cistus* extracts showed antibacterial activity against *S. aureus* ATCC 6538, methicillin-resistant *S. aureus* (MRSA) ATCC 43300, *S. epidermidis* ATCC 35984 and *P. acnes* ATCC 11827. In particular, *C. salviifolius* L. extracts revealed the highest antibacterial activity with MIC values of 62.5–125 μg/mL. None of the tested strains was inhibited by DMSO (maximum 1% *v*/*v*) used as negative control.

The determination of the killing activity revealed that the extracts possessed a bacteriostatic rather than bactericidal effect. In this context, the MBC values ranged from 1000 to >2000 μg/mL, except for *P. acnes,* which showed MBC values of 500–1000 μg/mL. These findings were confirmed by the MBC/MIC ratio of 4 for CSLEand CSLE PF against *P. acnes* (confirming a bactericidal effect) [21], and MBC/MIC ratio values higher than 4 for the other strains.

### 2.5. Cytotoxicity Evaluation

To characterize the cytotoxic effect of *Cistus* extracts, the viability of NIH/3T3 exposed for 48 h to different concentrations of the extracts was investigated. The findings indicated that the cytotoxic activity of the crude hydroalcoholic extracts was weaker with respect to the polyphenol fractions, suggesting that crude extracts are probably safer (Table 5).

## 3. Discussion

The management and the therapy of acne vulgaris aim to reduce sebum production, and follicular hyper keratinization. The drugs used should primarily exhibit antioxidant, anti-inflammatory, and antimicrobial activities, without having cytotoxic effects. Some synthetic remedies have limitations because of their highly toxic activity, and their ability to induce adverse side effects. Thus, a growing interest in the use of herbal medicines as coadjuvants for the treatment of acne is increasingly recognized. 

In our research, we assessed the composition and evaluated the biological properties of various extracts from the leaves of *Cistus laurifolius* L. and *C. salviifolius* L., grown in Morocco. Thus, we identified the main compounds present in both *Cistus* spp. and determined the antioxidant, anti-inflammatory, antimicrobial, and cytotoxic properties of such extracts (crude and polyphenolic fraction).

The *Cistus* genus was recently reviewed for its composition by Papaefthimiou et al. [22]. The authors reported *C. laurifolius* L. and *C. salviifolius* L. as a source of flavonoids, hydroxycinnamic acids, and tannins. In our study, 36 chromatographic signals were detected, tentatively identified, and quantified, as shown in Table 2. When considering the polyphenolic profile from the crude hydroalcoholic extracts of these two species (CLLE and CSLE in Table 2), at least two significant differences can be evidenced: the presence in CLLE of several hydroxycinnamic acids (mainly para-coumaric) derivatives (peaks 1, 3, 6, 8, and 12) as well as that of a bulky group of methyl- and polymethyl- flavonoids (peaks 22–26 and 28–36), that CSLE shares only to a minimum extent (Table 2). This particular richness in methyl-flavanoids of CLLE aligns with what reported in the literature [23]. On the other side, in CSLE, a series of ellagitannins were troublesomely identified, of which two couples of anomers (peaks 2 + 7 and 4 + 9) corresponding to terflavin A and cistusin, respectively, reported to belong to the secondary metabolism of *C. incanus* [24]. A gallic acid derivative (peak 5) and an ellagic acid derivative (peak 14) complete the ellagitannin series in CSLE. No punicalagin were detected, this time in disagreement with what was reported in the literature [15,16,17,18,19,20,21,22,23,24,25]. Regarding the quantitative analyses, small differences were detected between CLLE (3.74 mg polyphenols/100 mg extract) and CSLE (2.98 mg polyphenols/100 mg extract) (Table 2).

Therefore, polyphenols are mainly present in our extracts. Notably, the percentage of major compounds, calculated with respect to the total polyphenols reported in Table 2, was as follows: the derivatives of myricetin, quercetin, and hydroxycinnamic acids were, respectively 46%, 31%, and 7% for CLLE and 58%, 29%, and 3% for CLLE PF; while hydrolysable tannins were present in the CSL extracts, 51% for CSLE and 15% for CSLE PF, especially the ellagitannin cistusin, besides flavonoid derivatives (myricetin, quercetin and kaempferol) (Figure S2).

As well documented in the literature, the extracts of *C. laurifolius* L. and *C. salviifolius* L. showed important biological effects. In particular, a recent review by Zalegh et al. [26] described the antioxidant and anti-inflammatory effects of *Cistus* spp. extracts and their correlation with the polyphenolic content. Semwal et al. [27] reported that myricetin and its derivates are known for radical scavenging, immunomodulating and anti-inflammatory activities by interfering with the NF-kB signaling pathway and, as described by Imra et al. [28], the antioxidant activity of myricetin is attributed to the presence of three hydroxyl groups on ring B, as compared to other flavonoids. Furthermore, Lee and Lee [29] demonstrated that myricetin was able to exhibit differential anti-inflammatory effects on the production of inflammatory mediators in various cells and with distinctive stimulants.

Actually, quercetin is the one of the most powerful antioxidant flavonoids in nature. Recently, its biological activity was evaluated by Srimathi Priyanga and Vijayalakshmi [30] and Azeem et al. [31]. The authors highlighted the potential role in supporting the immune system against oxidative stress and its related disorder, the antimicrobial and the anti-inflammatory effects of this flavonoid.

Concerning tannins, de Melo et al. [32] described their ROS reduction characteristics. The authors reported that their activities are associated with the number and degree of polymerization of reactive hydroxyl groups present in the phenolic rings and that those characteristics are associated with anti-inflammatory, anti-aging, and health benefits. 

Therefore, the presence of myricetin and quercetin derivatives and hydrolysable tannins may partly explain the antioxidant and anti-inflammatory activities of the extracts under examination. 

Regarding antimicrobial activity, the best efficacy of our extracts was detected against Gram-positive bacteria such as *S. aureus*, including MRSA strain, *S. epidermidis*, and *P. acnes.* In contrast, the extracts were found to have a low activity on Gram-negative bacteria. This different effect can be partly due to the differences in cell wall structure among these microorganisms; the Gram-negative bacteria have an outer membrane that pro-vides an additional selective permeation barrier. 

The antimicrobial activity of some *Cistus* spp. has been explored. However, it is difficult to compare the data with the literature because different variables, such as the different collecting site, extraction solvent and antimicrobial method, can influence the results. *C. laurifolius* and *C. salviifolius* methanol extracts were reported to show activity against *Bacillus subtilis*, *B. cereus* and *S. aureus*, but no efficacy against *P. aeruginosa* and *C. albicans*. [33]. Tomàs-Menor et al. [25] investigated the relationship between the antimicrobial activity of *C. salviifolius* extracts and phytochemical composition. In this context, Zeouk et al. [34] reported that *C. salviifolius* methanol extracts with high amounts of total phenols and flavonoids showed antibacterial activity against MRSA and *S. epidermidis* strains. Moreover, Álvarez-Martínez et al. [35] investigated the activity of *C. salviifolius* against 100 *S. aureus* clinical isolates and referred MIC_50_ values ranging between 50–80 μg/mL. The authors also documented that the *Cistus* extracts containing hydrolysable tannins and flavonoids such as myricetin and quercetin derivatives showed the higher activity against MRSA isolates.Among the *Cistus* extracts, *C. laurifolius* exhibited antibacterial efficacy against *S. aureus* and *E. coli* [36] and inhibitory activity against microbial biofilm production [37]. 

Considering the literature and based on our results on antioxidant, anti-inflammatory, antimicrobial and citotoxic activities, we can say that *C. laurifolius* L. and *C. salvifolius* L. extracts possess a potential to treat mild acne.

## 4. Materials and Methods

### 4.1. Plant Material

Plant samples were collected at two different locations. The leaves of *C. laurifolius* L. were collected before and during plant flowering in March 2017, from Toufliht region in the High Atlas Mountains (70 km southeast of Marrakesh, Morocco). The leaves of *C. salviifolius* samples were collected during flowering in June 2017, from Moulay Bouzerktoun region, located on the Atlantic coast (30 km north of Essaouira, Morocco). Voucher specimens of *C. laurifolius* L. (MARK-8260) and *C. salviifolius* L. (MARK-14596) were deposited in the regional herbarium (MARK) of the Department of Biology, Faculty of Sciences Semelalia, Cadi ayyad University Marrakesh, Morocco.

### 4.2. Preparation of the Extracts 

**Figure 2:**
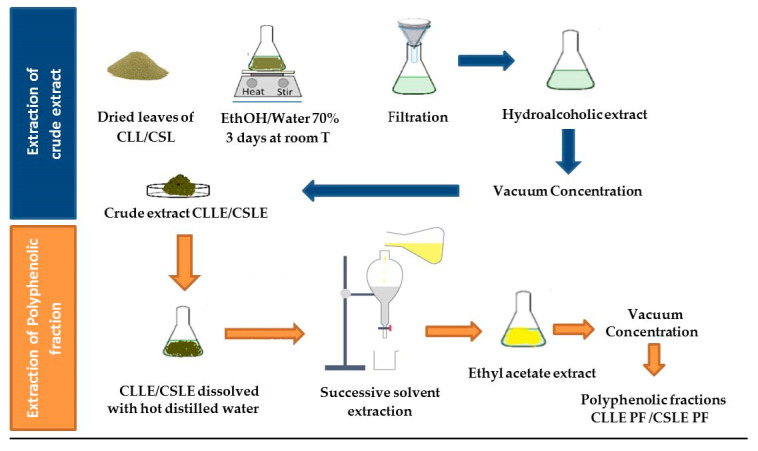
Extraction process of crude extracts and polyphenolic fractions of *C. laurifolius* L. and *C. salviifolius* L.

The powder of dried leaves of both plants was subjected to extraction with Ethanol/Water 70% (*v*/*v*) under permanent agitation at room temperature for 3 days. The obtained extracts were filtered and concentrated under reduced pressure to obtain dried residues of the ethanolic extracts (CLLE, CSLE) (Figure 2). The extraction yields are expressed in the percentage of dried plant material (Table 1).

As shown in Figure 2, the obtained hydroalcoholic crude extracts were dissolved in hot distilled water. They were processed with successive solvent extraction using three solvents with different polarities (n-hexane, dichloromethane, ethyl acetate). The ethyl acetate extract of each plant was filtered and evaporated under reduced pressure to obtain the respective polyphenolic-rich fractions (PF). 

### 4.3. Chemical Characterization

#### 4.3.1. Phytochemical Screening

Phytochemical analysis of the extracts was performed to determine the content of secondary metabolites such as total phenols, flavonoids, and flavonols. In all the tests, results were expressed as the mean ± SD of the three different experiments.

##### Total Polyphenol Content

The total polyphenol content of each extract was determined using the Folin–Ciocalteu reagent according to the method described by Tomaino et al. [38]. A volume of 0.5 mL of Folin–Ciocalteu reagent was mixed with 0.1 mL of each extract concentration previously diluted with distilled water (1:10). The solution was allowed to stand for 3 min at 25 °C before adding 1.7 mL of a 10% sodium carbonate solution (NA_2_CO_3_). The absorbance was measured at 786 nm after 1 h of incubation with agitation at room temperature. Total polyphenol content was determined using a standard curve prepared with gallic acid. The results are expressed as mg of gallic acid equivalents (GAE) per gram of dry extract (DE).

##### Flavonoids Content

The flavonoid content was measured using the method described by Bouaziz et al. [39]. Briefly, 1 mL of each sample at various concentrations ranging from 0.125 to 1 mg/mL, was mixed with 4 mL of distilled water, and 300 μL of a 5% sodium nitrate solution (NaNO_2_) was added. After 5 min, 300 µL of aluminum trichloride solution (AlCl_3_, 10%) was added to the mixture, followed 1 min later by the addition of 2 mL of sodium hydroxide (NaOH, 1 M). The absorbance was measured at 510 nm using a UV–VIS spectrophotometer. Total flavonoid content was calculated from a calibration curve using catechin as a standard and expressed as mg catechin equivalents (CatE) per g of DE.

##### Flavonols Content

The flavonols content of each extract was determined using the method described by Russo et al. [40]. Each extract (1 mL) at various concentration (0.125 to 1 mg/mL) was mixed with 1 mL of a 20% aluminum trichloride solution (AlCl_3_). Then, a volume of 3 mL of sodium acetate solution (C_2_H_3_NaO_2_, 50 mg/mL; *w*/*v*) was added. The mixed solutions were incubated in the dark for 2.5 h at room temperature. The absorbance was measured at 440 nm using a UV–Vis spectrophotometer. The content of flavonols was determined from a calibration curve, using quercetin as a standard. The results are expressed as mg of quercetin equivalents (QE) per g of dry extract.

#### 4.3.2. HPLC-DAD and HPLC-ESI-/MS Based Secondary Metabolic Profiles

Chromatographic analyses were carried out on an Ultimate3000 UHPLC focused instrument equipped with a binary high-pressure pump, a Photodiode Array detector, a Thermostated Column Compartment, and an Automated Sample Injector (Thermo Fisher Scientific, Inc., Milan, Italy). Collected data were processed through a Chromeleon Chromatography Information Management System v. 6.80. Chromatographic runs were performed using a reverse-phase column (Gemini C18, 250 × 4.6 mm, 5 μm particle size, Phenomenex Italia s.r.l., Bologna, Italy) equipped with a guard column (Gemini C18 4 × 3.0 mm, 5 μm particle size, Phenomenex Italia s.r.l., Bologna, Italy). Components of the hydroalcoholic extracts of *Cistus* spp. were eluted using a gradient of B (2.5% formic acid in acetonitrile) in A (2.5% formic acid in water): 0 min: 5% B; 10 min: 15% B; 30 min: 25% B; 35 min: 30% B; 50 min: 90% B; 57 min then kept for other 7 min, 100% B. The solvent flow rate was 1 mL/min, the temperature was kept at 25 °C, and the injector volume selected was 10 μL. Quantification was carried out at 330 nm using p-coumaric acid, ferulic acid, and apigenin commercial standards to build the calibration curves; for kaempferol, quercetin, and myricetin derivatives, the following standards were used at 350 nm: kaempferol 3-*O*-rutinoside, quercetin 3-*O*-glucoside, and myricetin; finally, punicalagin was used to calibrate instrument’s response for ellagitannins at 360 nm. In order to confirm peak assignments, HPLC/ESI-MS analyses were also performed. The HPLC apparatus was the same as described above, while ESI mass spectra were acquired by a Thermo Scientific Exactive Plu Orbitra MS (Thermo Fisher Scientific, Inc., Milan, Italy) using a heated electrospray ionization (HESI II) interface. Mass spectra were recorded operating in negative ion mode. Analyses were always carried out in triplicate.

### 4.4. Antioxidant Activity

The antioxidant effect was carried out through the following very well-known in vitro assays: 2, 2-diphenyl, 1-picrylhydrazyl radical scavenging (DPPH) assay, Ferric Reducing/Antioxidant Power (FRAP) assay and 2, 2′-azinobis [3-ethylbenzthiazoline]-6-sulfonic acid cation decolorization (ABTS) assay [41,42]. Results were expressed as mean ± SD of three different experiments carried out in triplicate.

### 4.5. Anti-Inflammatory Activity 

Anti-inflammatory activity was evaluated through the study of the ability of extracts to protect albumin (BSA) against HClO-induced denaturation. BSA was incubated in the absence or presence of 177 mM HClO and with increasing amounts of the extracts (0.05, 0.1, 0.25 mg/mL), then subjected to SDS-PAGE and developed with Coomassie Blue. Trolox at different concentrations (0.25, 0.088, and 0.062 mg/mL) was used as a positive/standard control [41]. Experiments were carried out in triplicate, and the results were expressed as IC50 values (mg extract/mL) and 90% C.L.

### 4.6. Antimicrobial Activity

#### 4.6.1. Microbial Strains and Culture Conditions

The antimicrobial activity was assessed against a panel of Gram-positive bacteria (*Staphylococcus aureus* ATCC 6538, methicillin-resistant *S. aureus* ATCC 43300, *Staphylococcus epidermidis* ATCC 35984, and *Propionibacterium acnes* ATCC 11827), Gram negative-bacteria (*Pseudomonas aeruginosa* DSM 102273, *Escherichia coli* ATCC 10536, *Klebsiella pneumoniae* DSM 26371) and yeast (*Candida albicans* ATCC 10231). Cultures were grown in Mueller-Hinton Broth (MHB) for 24 h (bacteria) and Sabouraud Dextrose Broth (SDB) for 48 h (yeast). *P. acnes* ATCC 11827 was grown in Brain–Heart Infusion Broth (BHI) under anaerobic conditions at 37 °C for 4 to 7 days.

#### 4.6.2. MIC and MBC/MFC Determination

The minimum inhibitory concentration (MIC), the minimum bactericidal concentration (MBC), and the minimum fungicidal concentration (MFC) of plant extracts were determined using a broth dilution micro method in 96-well microtiter plates according to Clinical and Laboratory Standards Institute (CLSI) guidelines with some modification [43,44]. Briefly, *Cistus laurifolius* L. and *Cistus salviifolius* L. extracts and their polyphenolic fractions were dissolved in pure dimethylsulfoxide (DMSO) to prepare respective stock solutions (200 mg/mL) from which serial twofold dilutions were made in MHB, BHI or SBD at final concentrations ranging from 2000 to 1.95 μg/mL. Microbial cultures were inoculated to yield a final concentration of 5 × 10^5^ CFU (bacteria) and 2.5 × 10^3^ CFU (yeast). All determinations were performed in duplicate and growth control consisting of MHB and MHB with DMSO 1% were included as controls. Ofloxacin and amphotericin B were used as positive reference standard drugs. The MIC was considered as the lowest concentration of each extract, giving a complete inhibition of visible bacterial growth after incubation for 24 h (bacteria), 48 h (yeast), and in anaerobic conditions for 4 to 5 days (*P. acnes*). The MBC was determined by seeding 20 μL from all clear MIC wells onto agar plates and was defined as the lowest concentration of plant extracts that killed 99.9% of the inoculum. Each determination was performed in three independent experiments, and modal results were calculated.

### 4.7. Cytotoxicity Evaluation

The biocompatibility of both plant extracts was assessed on NIH/3T3 fibroblasts cell line (ATCC CRL-1658; American Type Culture Collection, Rockville, MD, USA) using the Sulforhodamine B (SRB) assay. The NIH/3T3 cells were exposed for 48 h to different concentrations (15.5, 31, 62.5, and 125 µg/mL) of tested extracts added to the cell culture medium, or to the vehicle alone (DMSO) used as the control [45]. 

### 4.8. Statistical Analysis 

Results are statistically analyzed by a one-way or a two-way analysis of variance (ANOVA) test, followed by Tukey’s honest significant difference, using the statistical software ezANOVA (https://people.cas.sc.edu/rorden/ezanova/index.html, accessed on 28 February 2023)

## 5. Conclusions

Overall, the results suggest that the crude extracts of *Cistus laurifolius* L. and *C. salviifolius* L. from the High Atlas Mountain of Morocco, despite the slightly different activity, can be regarded as promising agents to develop coadjuvants/natural remedies for the treatment of mild acne, and it is worth noting that extracts could be subjected to in vivo study.

## Figures and Tables

**Table 1:** Phytochemical analysis and yield extraction of *C. laurifolius* L. and *C. salviifolius* L. crude extracts and their polyphenol fractions.

	Crude Extracts		Polyphenol Fractions	
	CLLE	CSLE	CLLE PF	CSLE PF
Yield %	15.8 ± 0.8	15.2 ± 1.0	0.9 ± 0.1	1.0 ± 0.1
Total polyphenol content mgGAE/g DE	110.2 ± 59.7	258.4 ± 28.2	419.8 ± 9.2	456.1 ± 24.4
Flavonoids mgCatE/g DE	113.7 ± 32.4	111.9 ± 11.8	172.2 ± 26.6	144.4 ± 10.3
Flavonols mgQE/g DE	56.2 ± 5.2	33.9 ± 0.5	123.4 ± 6.4	114.7 ± 14.4

CLLE = *Cistus laurifolius* L. hydroalcoholic extract; CSLE = *Cistus salviifolius* L. hydroalcoholic extract; PF = polyphenol fractions; GAE: Gallic acid equivalents; CatE: catechin equivalents; QE: quercetin equivalents; DE: dry extracts; PF; polyphenol fraction. Results are expressed as means ± SD of three different experiments.

**Table 2:** Content of individual metabolites from the *Cistus* spp. extracts object of this study, reported in mg over 100 g of extract.

Peak	Compound	CLLE	CSLE	CLLE PF	CSLE PF
1	P-coumaroyl quinic acid, isomer 1	0.0337	n.d. *	n.d.	n.d.
2	Terflavin A anomer 1	n.d.	0.6234	n.d.	0.2343
3	P-coumaroyl quinic acid, isomer 2	0.0385	n.d.	n.d.	n.d.
4	Cistusin anomer 1	n.d.	0.0732	n.d.	0.3984
5	Gallagic acid derivative	n.d.	0.5044	n.d.	0.5476
6	P-coumaroyl glucose, isomer 1	0.0560	n.d.	0.0403	n.d.
7	Terflavin A anomer 2	n.d.	0.0537	n.d.	0.0271
8	P-coumaroyl glucose, isomer 2	0.0864	n.d.	0.0641	n.d.
9	Cistusin anomer 2	n.d.	0.1247	n.d.	0.2652
10	Myricetin hexoside derivative	1.4631	0.2132	8.7267	2.6069
11	Quercetin derivative	0.0534	0.0114	0.3354	0.1971
12	Feruloyl glucose	0.0540	0.0253	0.4464	n.d.
13	Myricetin hexoside	0.2351	0.1822	2.5111	2.9201
14	Ellagic acid galloyl hexoside	n.d.	0.1415	n.d.	1.5511
15	Rutin *	0.3328	0.2804	3.0759	2.9646
16	Quercetin 3-*O*-glucoside *	0.0823	0.2332	0.9527	2.8943
17	Quercetin 3-*O*-rhamnoside *	n.d.	0.0971	n.d.	1.1118
18	Kaempferol hexoside	0.0176	0.0164	0.2496	0.2359
19	Kaempferol 3-*O*-glucoside *	0.0085	n.d.	0.1429	n.d.
20	Myricetin *	0.0366	n.d.	0.1950	0.6625
21	Luteolin hexoside-deoxyhexoside	0.0267	n.d.	0.1111	n.d.
22	Methyl-quercetin	0.3051	n.d.	1.1705	0.4556
23	Methyl kaempferol, isomer 1	0.1902	0.4046	1.1919	2.9671
24	Methyl kaempferol, isomer 2	0.0434	n.d.	0.0681	n.d.
25	Methyl kaempferol, isomer 3	0.0120	n.d.	0.0129	n.d.
26	Di-methyl quercetin isomer 1	0.1482	n.d.	0.0835	n.d.
27	Luteolin *	0.0172	n.d.	0.0076	n.d.
28	Di-methyl quercetin isomer 2	0.1883	n.d.	0.1021	n.d.
29	Di-methyl quercetin isomer 3	0.0308	n.d.	0.0080	n.d.
30	Methyl apigenin isomer 1	0.0395	n.d.	0.0123	n.d.
31	Methyl apigenin isomer 2	0.1017	n.d.	0.0294	n.d.
32	Di-methyl kaempferol	0.0448	n.d.	0.0059	n.d.
33	Di-methyl quercetin derivative	0.0298	n.d.	n.d.	n.d.
34	Methyl luteolin	0.0217	n.d.	n.d.	n.d.
35	Di-methyl apigenin	0.0322	n.d.	0.0117	n.d.
36	Di-methyl kaempferol	0.0074	n.d.	n.d.	n.d.
***Total polyphenols***		***3.7371 ± 0.0017***	***2.9846 ± 0.0017***	***19.555 ± 0.0034***	***20.039 ± 0.0001***
*Total flavonoids*		*3.4685 ± 0.0016*	*1.4385 ± 0.0012*	*19.004 ± 0.0029*	*17.017 ± 0.0002*
*Total hydroxycinnamic acids and derivatives*		*0.2686 ± 0.0004*	*0.0253 ± 0.0003*	*0.5508 ± 00005*	*-*
*Total tannins*		*-*	*1.5208 ± 0.0004*	*-*	*3.0236 ± 0.0003*

CLLE = *Cistus laurifolius* L. hydroalcoholic extract; CSLE = *Cistus salviifolius* L. hydroalcoholic extract; PF = polyphenol fractions; n.d. = not detected; * identified with the help of the pure commercial standard.

**Table 3:** Scavenging activity evaluation of *C. laurifolius* L. and *C. salviifolius* L. crude extracts and their polyphenolic fractions.

	Crude Extracts		Polyphenolic Fractions	
Tests	CLLE	CSLE	CLLE PF	CSLE PF
DPPH (mmol TE/mg DE)	0.551 ± 0.044	0.639 ± 0.046	1.284 ± 0.327	2.593 ± 0.572 ^a^
ABTS (mmol TE/mg DE)	2.072 ± 0.532	3.267 ± 0.776	2.668 ± 0.433	2.668 ± 0.433
FRAP (mmol Fe^2+^E/mg DE)	2.370 ± 0.290	8.41 ± 0.26	9.840 ± 0.620	11.520 ± 1.150 ^bc^

CLLE = *Cistus laurifolius* L. hydroalcoholic extract; CSLE = *Cistus salviifolius* L. hydroalcoholic extract; PF = polyphenol fractions. TE: Trolox equivalents; Fe^2+^ E: ferrous equivalent; DE: dry extracts. Results are expressed as means ± SD of three different experiments. ^a^ *p* < 0.01 vs. all other extracts; ^b^ *p* < 0.05 vs. CSLE; ^c^ *p* < 0.01 vs. CLLE.

**Table 4:** BSA protection against HClO-induced degradation by *C. laurifolius* L. and *C. salviifolius* L. crude extracts and their polyphenolic fractions.

Extract	IC_50_ mg/mL
CLLE	0.126 (0.109–0.146)
CSLE	0.067 (0.054–0.084) ^a^
CLLE PF	0.103 (0.087–0.121)
CSLE PF	0.042 (0.033–0.052) ^a^
Trolox	0.05 (0.044–0.056)

CLLE = *Cistus laurifolius* L. hydroalcoholic extract; CSLE = *Cistus salviifolius* L. hydroalcoholic extract; PF = polyphenol fractions. Results are expressed as IC_50_ values (mg extract/mL) and 90% C.L. ^a^ *p* < 0.05 vs. CLLE and CLLE PF.

**Table 5:** Minimum inhibitory concentration (MIC), minimum bactericidal concentration (MBC) and minimum fungicidal concentration (MFC) of crude extracts and polyphenolic fractions of both *Cistus* species, and LC_50_ with 90% confidence limits (C.L.) at 48 h on 3T3 cell line.

	CLLE		CSLE		CLLE PF		CSLE PF	
Microbial Strains	MIC (µg/mL)	MBC (µg/mL)	MIC (µg/mL)	MBC (µg/mL)	MIC (µg/mL)	MBC (µg/mL)	MIC (µg/mL)	MBC (µg/mL)
*S. aureus* *ATCC 6538*	250	2000	125	2000	250	2000	125	2000
*S. aureus* *ATCC 43300*	250	2000	125	2000	250	2000	125	2000
*S. epidermidis* *ATCC 35984*	125	2000	62.5	2000	125	2000	62.5	2000
*P. acnes* *ATCC 11827*	125	1000	125	500	125	1000	125	500
*P. aeruginosa* *DSM 102273*	1000	>2000	500	>2000	1000	>2000	500	>2000
*E. coli* *ATCC 10536*	1000	>2000	1000	>2000	1000	>2000	1000	>2000
*K. pneumoniae* *DSM 26371*	2000	>2000	1000	>2000	2000	>2000	1000	>2000
*C. albicans* *ATCC 10231*	2000	>2000	500	>2000	2000	>2000	500	>2000
**LC_50_ (C.L. 95) at 48 h in NIH/3T3 cells (µg/mL)**	**171 (140–210)**		**>250**		**78 (65–93)**		**98 (90–108)**	

CLLE = *Cistus laurifolius* L. hydroalcoholic extract; CSLE = *Cistus salviifolius* L. hydroalcoholic extract; PF = polyphenol fractions.

## Data Availability

Not applicable.

## Supplementary Materials

The following supporting information can be downloaded at: https://www.mdpi.com/article/10.3390/molecules28062797/s1, Figure S1: HPLC/DAD chromatograms, visualized at 330 nm, of the *Cistus* spp. crude extracts object of this study. Peak numbers correspond to Table 2.; Figure S2: Composition (%) in polyphenols, divided into biochemical subclasses, of the *Cistus* spp. extracts. See text for details.; Table S1: Peak list and diagnostics of selected metabolites from the *Cistus* spp. extracts object of this study. See text for details.

## Sample Availability

Samples of the compounds are not available from the authors.
