# Supplementary material for: Efficacy of Two Moroccan Cistus Species Extracts against Acne Vulgaris: Phytochemical Profile, Antioxidant, Anti-Inflammatory and Antimicrobial Activities
Source: Molecules. 2023 Mar 20;28(6):2797. doi: 10.3390/molecules28062797 (PMC10054591; doi:10.3390/molecules28062797)
Supplement: Supplementary file 1 [file molecules-28-02797-s001.zip › molecules-2283495-supplementary.pdf]

Table S1. Peak list and diagnostics of selected metabolites from the *Cistus* spp. extracts object of this study. See text for details.

Peak	Rt, min[a]	compound tentative identification	UV-vis data, nm[b]	MW	ESI$^-$ data, m/z[c]
1	6,7	p-coumaroyl quinic acid, isomer 1	314, 298sh	338	337*(M−H), 146
2	7,2	terflavin A anomer 1	258, 377	1086	1085(M−H) (tr)
3	8,4	p-coumaroyl quinic acid, isomer 2	312, 299sh	338	337*(M−H)
4	8,4	cistusin anomer 1	258, 382	1252	1251(M−H) (tr)
5	9,3	gallagic acid derivative	258, 380	800	799 (M−H), 637*
6	9,9	p-coumaroyl glucose, isomer 1	311, 298sh	326	325*(M−H)
7	10,2	terflavin A anomer 2	258, 379	1086	1085*(M−H)
8	10,6	p-coumaroyl glucose, isomer 2	314, 290sh	326	325*(M−H)
9	11,2	cistusin anomer 2	258, 381	1252	1251(M−H)
10	17,6	myricetin hexoside derivative	260,356	656	655(M−H), 493*
11	18,0	quercetin derivative	256,354	626	625(M−H), 611*
12	19,1	feruloyl glucose	324, 293sh	356	355*(M−H)
13	20,3	myricetin hexoside	261,356	480	479*(M−H)
14	21,0	ellagic acid galloyl hexoside	252,367	616	615(M−H), 453*
15	21,5	rutin[d]	255, 353	610	609*(M−H)
16	24,5	quercetin 3-*O*-glucoside[d]	255, 354	464	463*(M−H)
17	25,1	quercetin 3-*O*- rhamnoside[d]	255, 352	448	447*(M−H)
18	26,0	kaempferol hexoside	264, 348	448	447*(M−H), 285
19	28,8	kaempferol 3-*O*-glucoside[d]	265, 347	448	447*(M−H)
20	35,9	myricetin[d]	254, 370	318	317*(M−H)
21	36,9	luteolin hexoside-deoxyhexoside	253, 266, 345	594	593(M−H),431*
22	38,2	methyl-quercetin	255, 355	316	315*(M−H)
23	38,7	methyl kaempferol, isomer 1	266, 314	300	299*(M−H)
24	41,0	methyl kaempferol, isomer 2	266, 341	300	299 (M−H),599 (2M−H)
25	41,8	methyl kaempferol, isomer 3	267, 346	300	299*(M−H)
26	42,1	di-methyl quercetin isomer 1	254, 354	330	329(M−H), 315*
27	43,1	luteolin[d]	253, 264, 345	286	285*(M−H)
28	43,5	di-methyl quercetin isomer 2	255, 354	330	329*(M-H)
29	44,1	di-methyl quercetin isomer 3	253, 354	330	329* (M−H), 659(2M−H)
30	44,8	methyl apigenin isomer 1	268, 326	284	283 (M−H), 269*
31	44,9	methyl apigenin isomer 2	267, 334	284	283*(M−H)
32	45,4	di-methyl kaempferol	266, 345	314	313 (M−H), 299*
33	45,6	di-methyl quercetin derivative	254, 353	390	389*(M−H), 779

34	45,7	methyl luteolin	255, 266, 354	300	299*(M−H)
35	48,1	di-methyl apigenin	269, 330	300	299 (M−H), 269*
36	48,6	di-methyl kaempferol	267, 346	314	313 (M−H), 299, 285*

[a] as mean of three replicates; [b] from HPLC; [c] base peaks marked with an asterisk; [d] co-injection with pure commercial standard

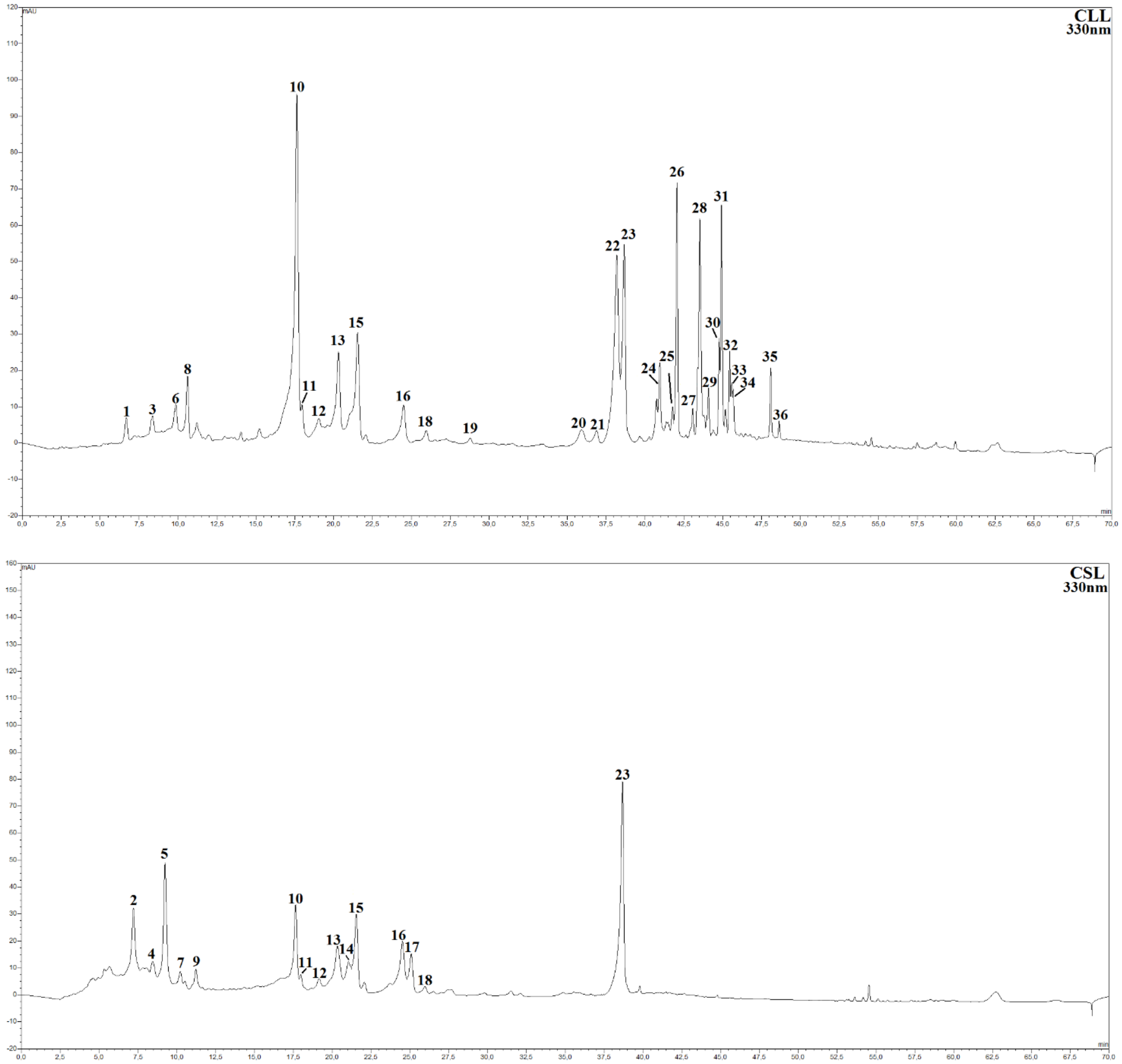

Figure S1. HPLC/DAD chromatograms, visualized at 330nm, of the *Cistus* spp. crude extracts object of this study. Peak numbers correspond to table 2.

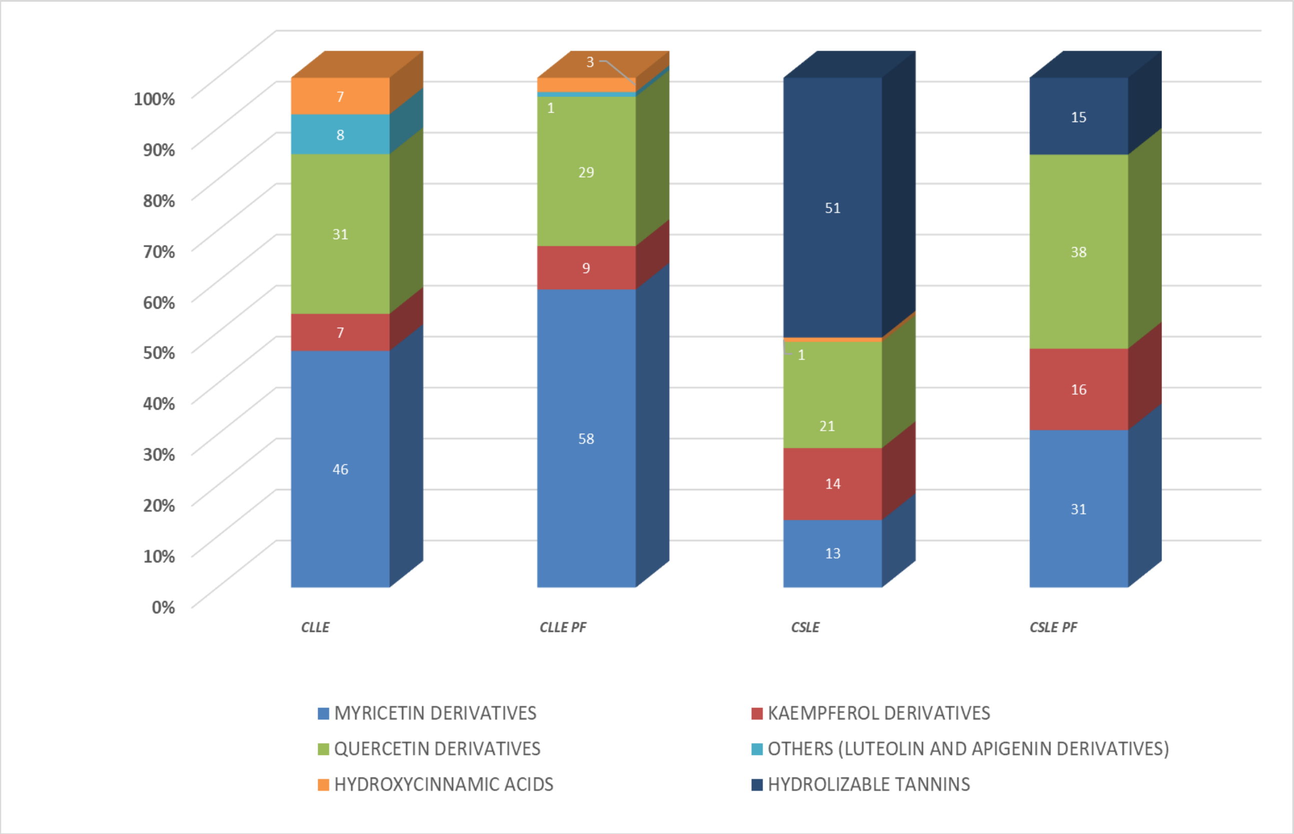

Figure S2. Composition (%) in polyphenols, divided into biochemical subclasses, of the *Cistus* spp. extracts. See text for details.
